# Supplementary figures and images for: First DNA barcode library for the ichthyofauna of the Jos Plateau (Nigeria) with comments on potential undescribed fish species
Source: PeerJ. 2022 Apr 13;10:e13049. doi: 10.7717/peerj.13049 (PMC9013235; doi:10.7717/peerj.13049)

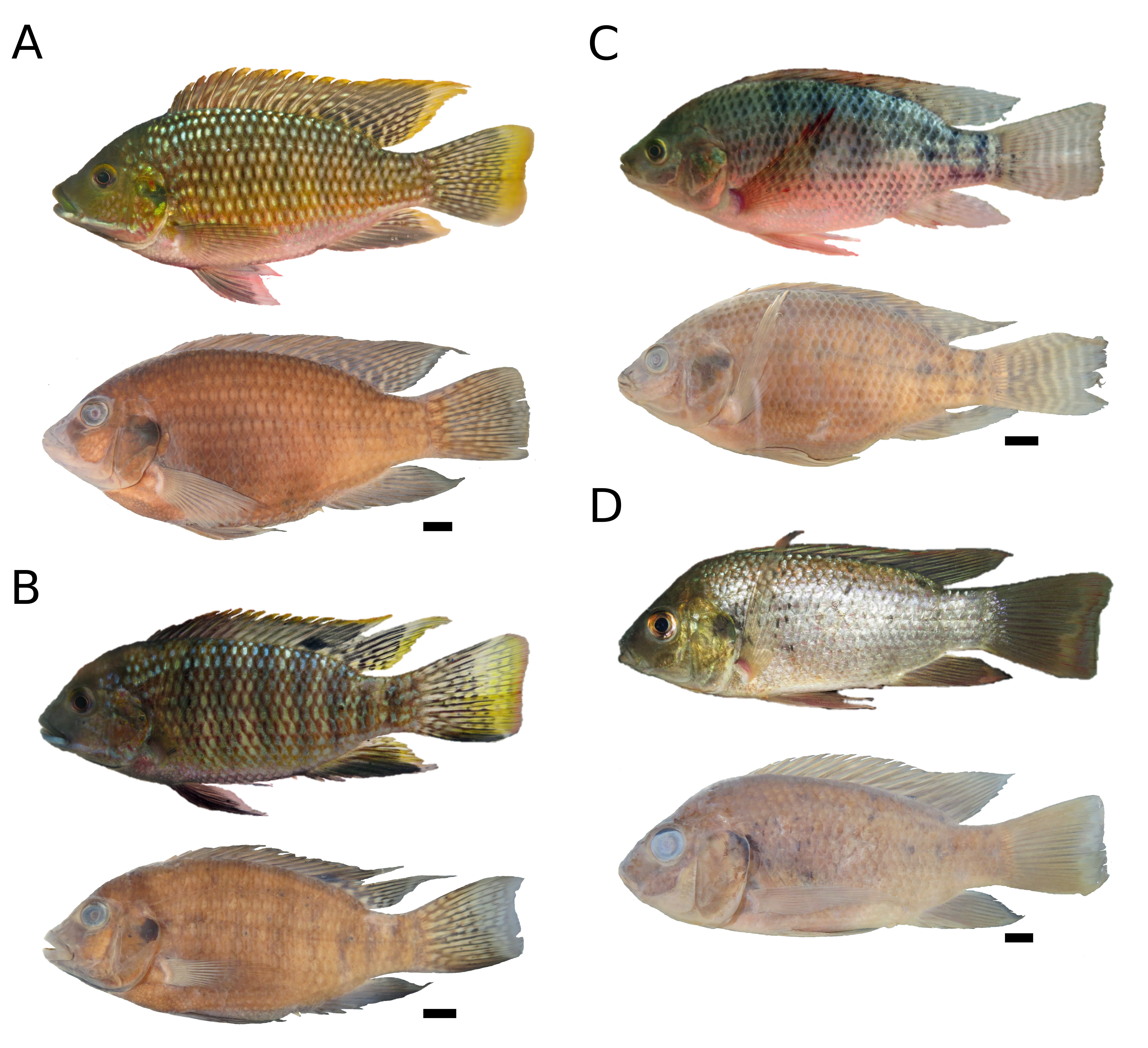

Supplement: Supplemental Information 1 — Photo credit: Popoola Michael Olaoluwa [file peerj-10-13049-s001.png]

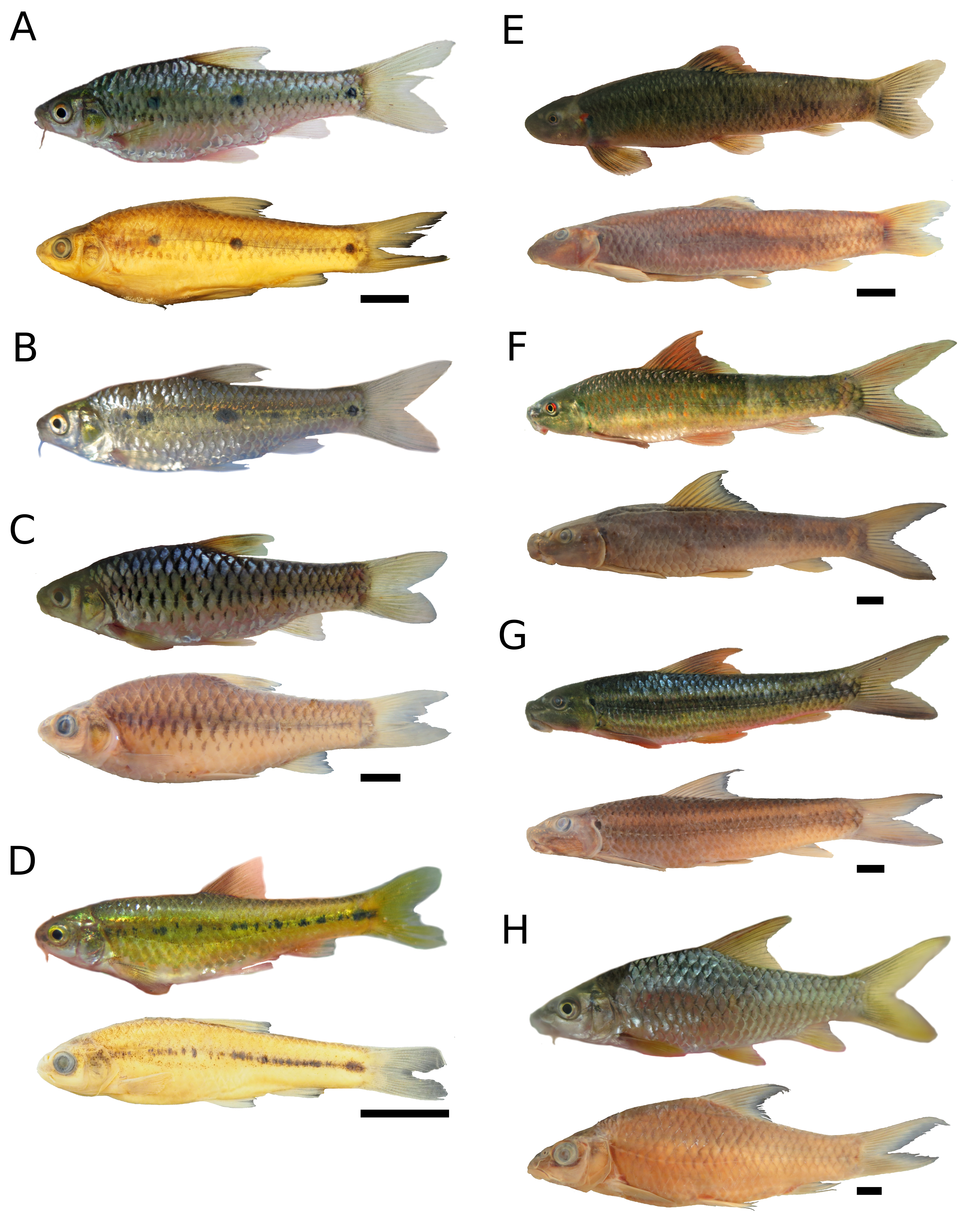

Supplement: Supplemental Information 2 — Photo credit: Popoola Michael Olaoluwa [file peerj-10-13049-s002.png]

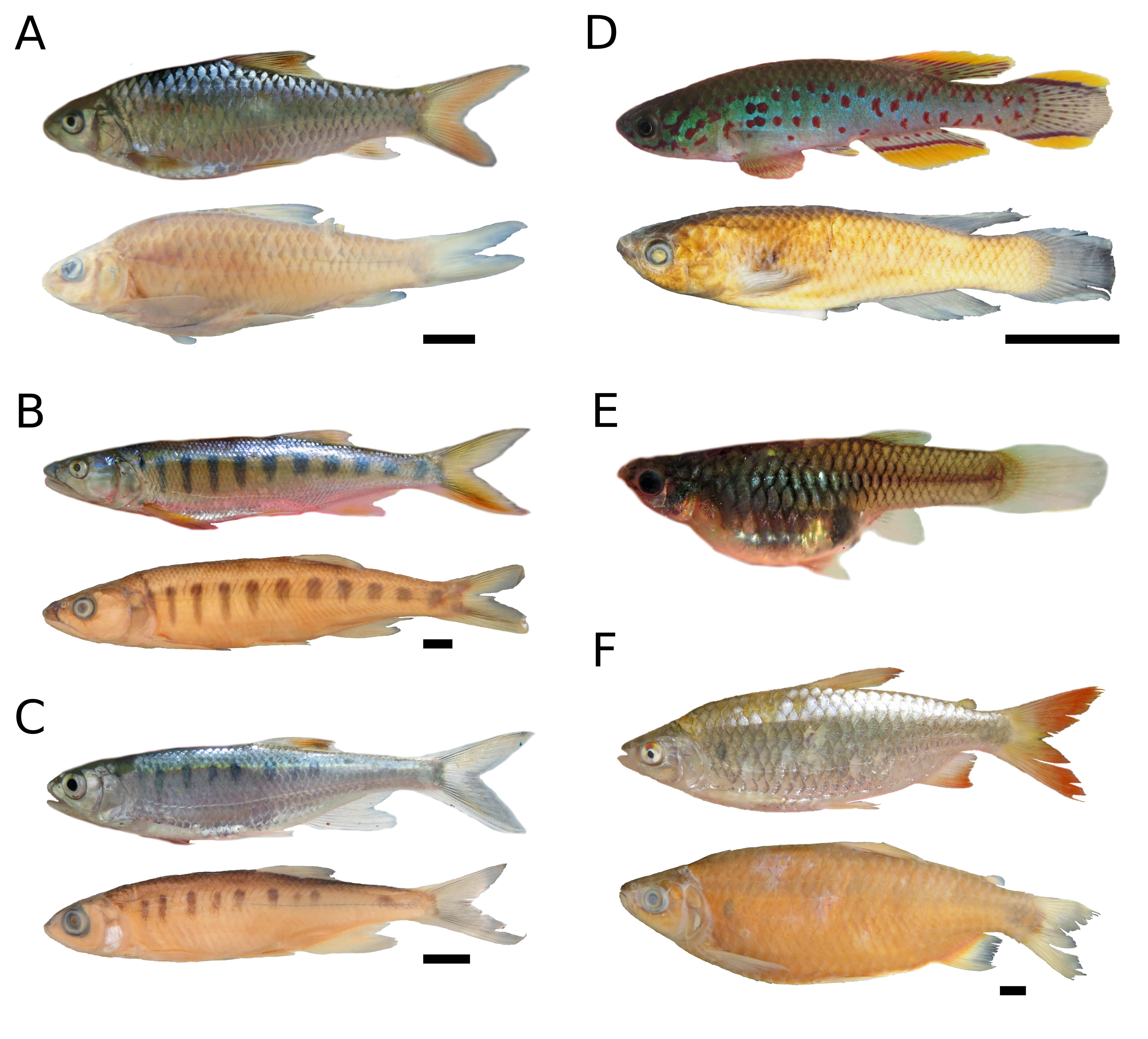

Supplement: Supplemental Information 3 — Photo credit: Popoola Michael Olaoluwa [file peerj-10-13049-s003.png]

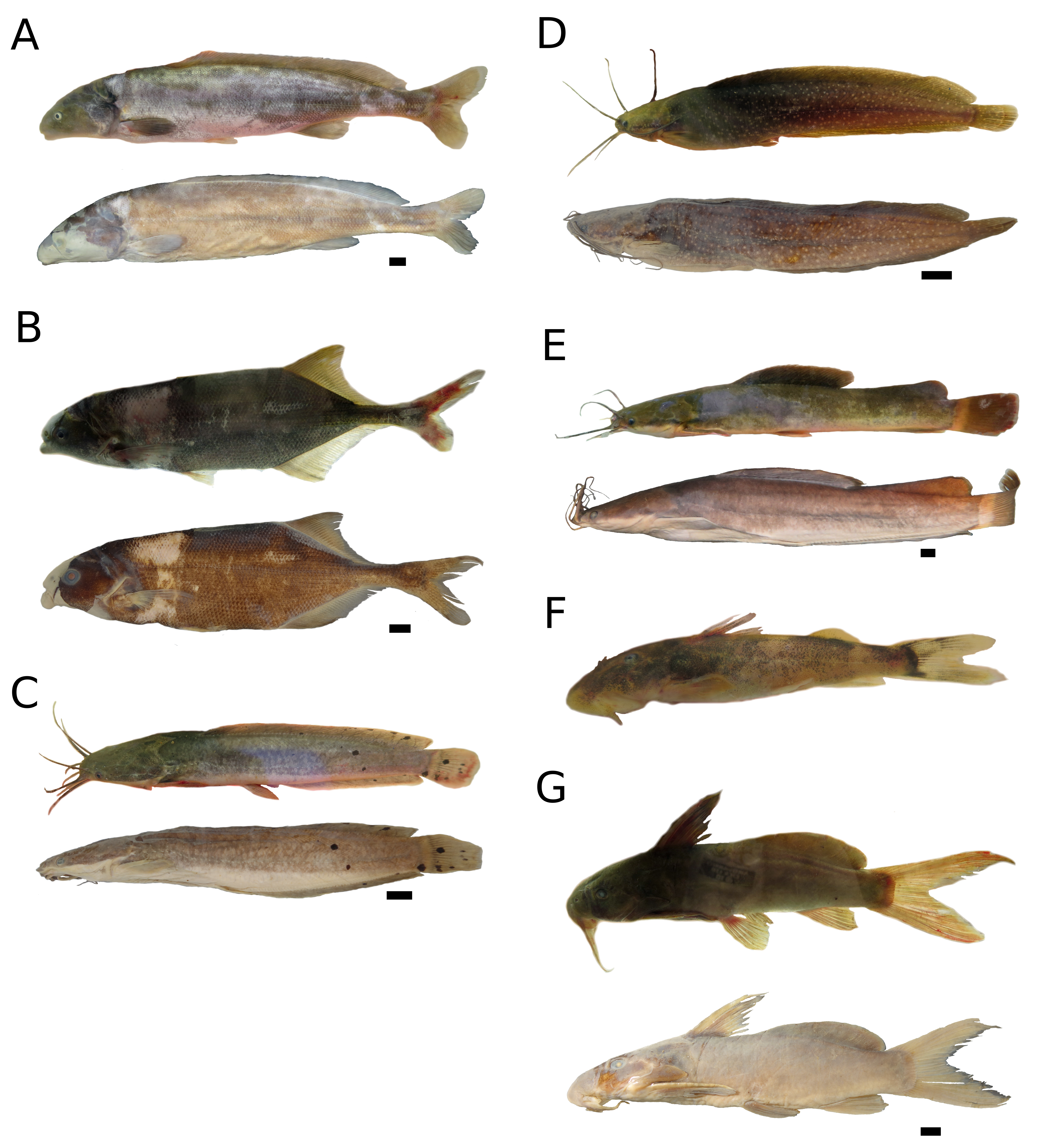

Supplement: Supplemental Information 4 — Photo credit: Popoola Michael Olaoluwa [file peerj-10-13049-s004.png]
