# Supplementary material for: First DNA barcode library for the ichthyofauna of the Jos Plateau (Nigeria) with comments on potential undescribed fish species
Source: PeerJ. 2022 Apr 13;10:e13049. doi: 10.7717/peerj.13049 (PMC9013235; doi:10.7717/peerj.13049)

Neighbour joining tree based on all available CO1 sequences on BOLD of the genus *Enteromius* (481 sequences representing 108 BINs), created in BOLD using "Taxon ID tree" .

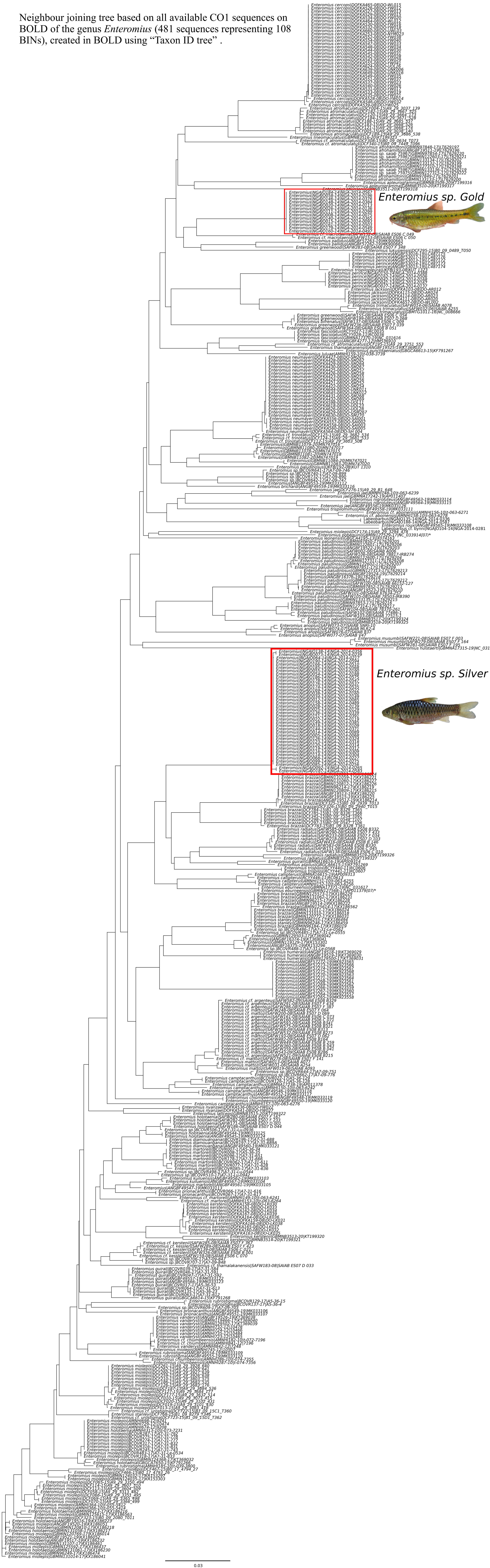

Supplement: Supplemental Information 5 [file peerj-10-13049-s005.pdf]
