# Supplementary material for: First DNA barcode library for the ichthyofauna of the Jos Plateau (Nigeria) with comments on potential undescribed fish species
Source: PeerJ. 2022 Apr 13;10:e13049. doi: 10.7717/peerj.13049 (PMC9013235; doi:10.7717/peerj.13049)

Neighbour joining tree based on all available CO1 sequences on BOLD of the genus *Clarias* (526 sequences representing 35 BINs), created in BOLD using "Taxon ID tree" tool and using Kaling (Lassmann and Sonnhammer, 2005).

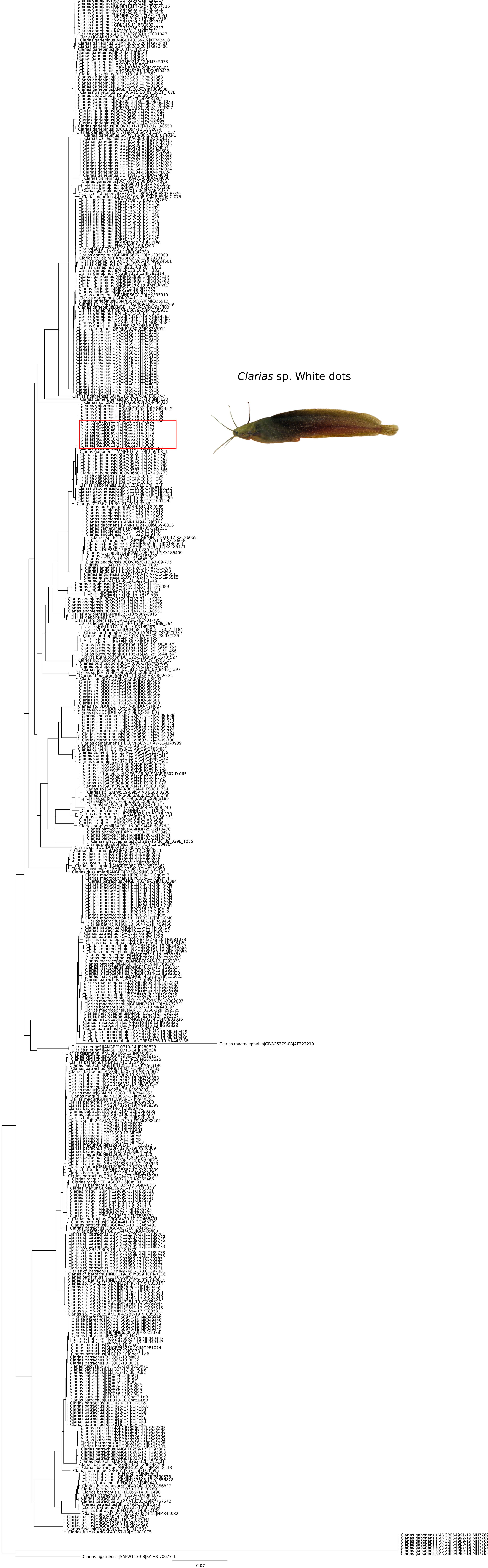

Supplement: Supplemental Information 7 [file peerj-10-13049-s007.pdf]
