# Supplementary material for: First DNA barcode library for the ichthyofauna of the Jos Plateau (Nigeria) with comments on potential undescribed fish species
Source: PeerJ. 2022 Apr 13;10:e13049. doi: 10.7717/peerj.13049 (PMC9013235; doi:10.7717/peerj.13049)
Supplement: Supplemental Information 8 — Only the clusters close to Labeo sp. Asop are shown. [file peerj-10-13049-s008.pdf]

Neighbour joining tree based on all available CO1 (incl. data minded from GenBank) sequences on BOLD of the genus *Labeo* (1393 sequences representing 78 BINs), created in BOLD using “Taxon ID tree”. Only the clusters close to *Labeo* sp. Asop are shown.

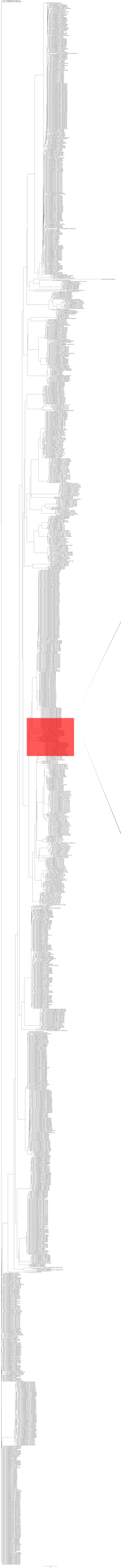

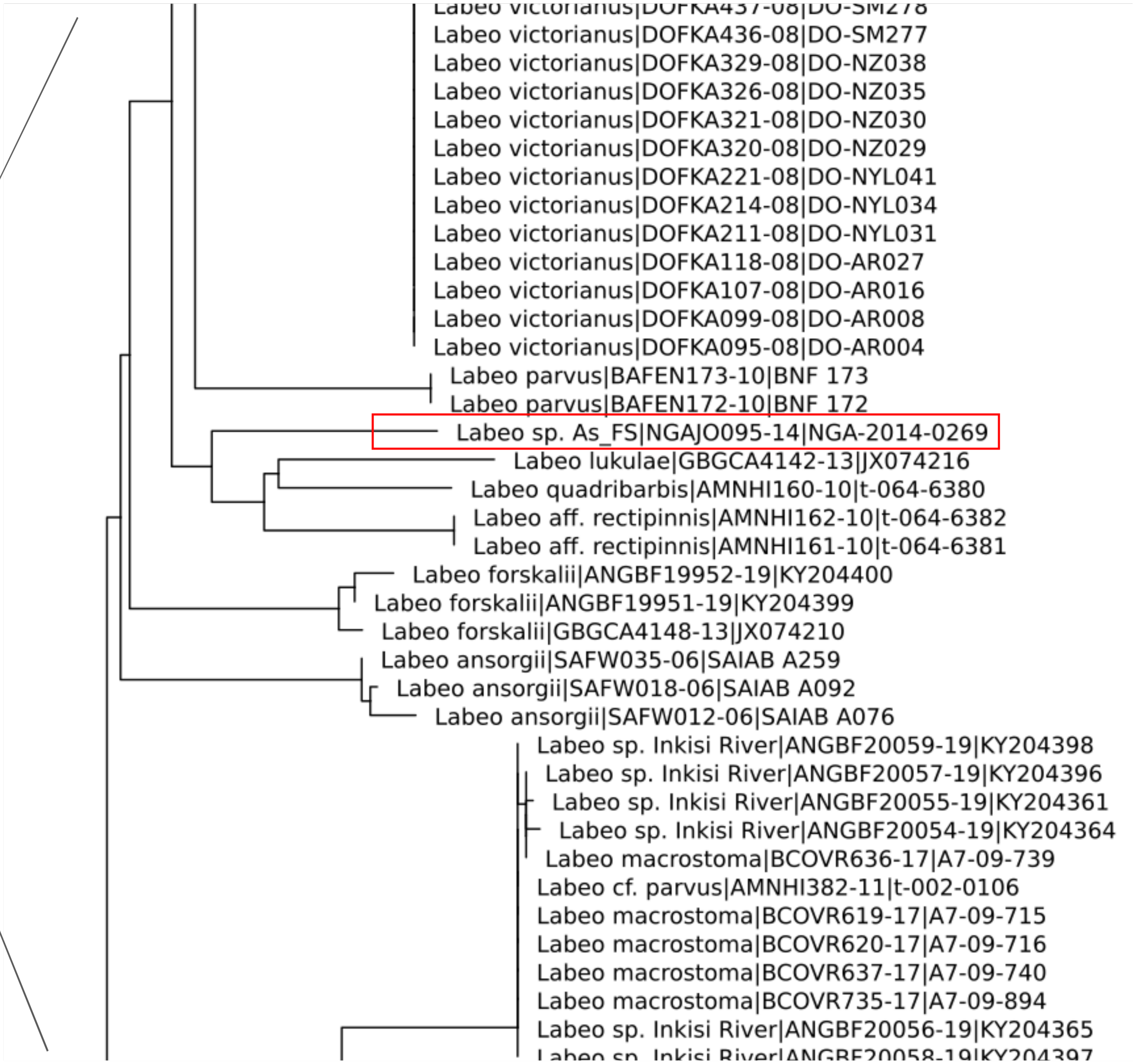

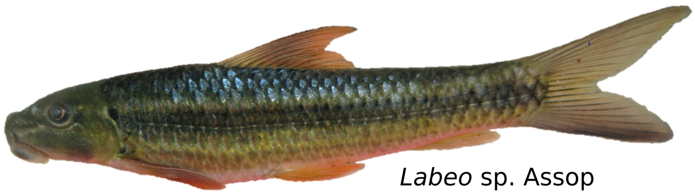

Labeo sp. Assop
