# Supplementary material for: First DNA barcode library for the ichthyofauna of the Jos Plateau (Nigeria) with comments on potential undescribed fish species
Source: PeerJ. 2022 Apr 13;10:e13049. doi: 10.7717/peerj.13049 (PMC9013235; doi:10.7717/peerj.13049)
Supplement: Supplemental Information 9 [file peerj-10-13049-s009.docx]

| **Locality Number** | **Sample location (River Name):** | **Further information** | **Latitude (North/South)** | **Longitude (West/East)** | **Altitude:** | **Drainage information:** |
| --- | --- | --- | --- | --- | --- | --- |
| 1. | Assop | Before Assop waterfalls, at Hawan Kibo | 9.5022 | 8.626067 | 762 m | Affluent of Mada River / Benue basin |
| 2. | Magurji | After Kura waterfalls, at Hawan Kibo | 9.504 | 8.619083 | 718 m | Affluent of Mada River / Benue basin |
| 3. | Tahoss | 7 (1+2+3) A congruence of Tahoss 1, 2, and 3 | 9.610433 | 8.7115 | 1138 m | Affluent of Mada River / Benue basin |
| 4. | Assop | below the water fall | 9.519033 | 8.609267 | 726 m | Affluent Mada River / Benue basin |
| 5. | Tahoss | 5 | 9.60315 | 8.689833 | 1104 m | Affluent of Mada River / Benue basin |
| 6. | Tahoss | 1 | 9.61305 | 8.716767 | 1147 m | Affluent of Mada River / Benue basin |
| 7. | Tahoss | 6 (1+2) A congruence of Tahoss 1 and 2 | 9.612133 | 8.716183 | 1162 m | Affluent of Mada River / Benue basin |
| 8. | Tahoss | 2 | 9.6159 | 8.7183 | 1173 m | Affluent of Mada River / Benue basin |
| 9. | Tahoss | 3 | 9.612067 | 8.7139 | 1150 m | Affluent of Mada River / Benue basin |
| 10. | Tahoss | 4 | 9.601467 | 8.708633 | 1131 m | Affluent of Mada River / Benue basin |
| 11. | N'gell | Before the bridge on the Jos-Bukuru express road | 9.829167 | 8.87285 | 1214 m | Affluent to Kaduna river |
| 12. | N'gell | After the bridge on the Jos-Bukuru express road | 9.808183 | 8.8484 | 1211 m | Affluent to Kaduna river |
| 13. | N'gell | After the second bridge along the river course the one on the Jos-Bukuru express road being the first bridge | 9.805133 | 8.847133 | 1213 m | Affluent to Kaduna river |
| 14. | N'gell | At Kwall after the Kwall waterfalls | 9.838517 | 8.644567 | 828 m | Affluent to Kaduna river |
| 15. | Gurum | Downstream of the river | 10.026333 | 8.83195 | 1122 m | Northern affluent of Jos Plateau and tributary to Karami and Kaduna rivers |
| 16. | Gurum | Upstream of the river | 9.993167 | 8.825833 | 1146 m | Northern affluent of Jos Plateau and tributary to Karami and Kaduna rivers |
| 17. | Gurum | Upstream of the river | 9.995883 | 8.827683 | 1112 m | Northern affluent of Jos Plateau and tributary to Karami and Kaduna rivers |
| 18. | Shen Fusa | Close to the dam | 9.770333 | 8.94745 | 1231 m | Trib. to eastern affluents Maijuju and Gongola Rivers of Jos Plateau, Benue subbasin |
| 19 | Shen Fusa | At Rett | 9.776583 | 8.931433 | 1250 m | Trib. to eastern affluents Maijuju and Gongola Rivers of Jos Plateau, Benue subbasin |
| 20. | Kassa | 1 | 9.55885 | 8.9054 | 1268 m | Trib. to eastern affluents Maijuju and Gongola Rivers of Jos Plateau, Benue subbasin |
| 21. | Kassa | 2 | 9.55175 | 8.901717 | 1276m | trib. to eastern affluents Maijuju and Gongola Rivers of Jos Plateau, Benue subbasin |
| 22. | Rukuba | 1 | 9.944967 | 8.77865 | 1197 m | Affluent to Kaduna river |
| 23. | Rukuba | 2 | 9.95995 | 8.771 | 1174 m | Affluent to Kaduna river |
| 24. | Foron | 2 | 9.69305 | 8.88705 | 1234 m | Trib. to eastern affluents Maijuju and Gongola Rivers of Jos Plateau, Benue subbasin |
| 25. | Foron | 1 | 9.695133 | 8.8853 | 1232 m | Trib. to eastern affluents Maijuju and Gongola Rivers of Jos Plateau, Benue subbasin |
| 26. | Gindiri | 1 | 9.598733 | 9.222567 | 1232 m | Trib. to eastern affluents Maijuju and Gongola Rivers of Jos Plateau, Benue subbasin |
| 27. | Gindiri | Mangu village | 9.6072 | 9.218667 | 1015 m | Trib. to eastern affluents Maijuju and Gongola Rivers of Jos Plateau, Benue subbasin |
| 28. | Delimi | 1 | 9.959967 | 8.87455 | 1136 m | Trib. to northeastern Bunga/Jama'are affluent of Jos Plateau, Komadugu-Yobe subbasin |
| 29. | Delimi | Babale village | 10.028367 | 8.940867 | 896 m | Trib. to northeastern Bunga/Jama'are affluent of Jos Plateau, Komadugu-Yobe subbasin |
| 30. | Jarawa | 1 | 9.98055 | 9.110417 | 950 m | Trib. to eastern affluents Bagel and Gongola Rivers of Jos Plateau, Benue subbasin |
| 31. | Jarawa | 2 | 9.987783 | 9.084567 | 943 m | Trib. to eastern affluents Bagel and Gongola Rivers of Jos Plateau, Benue subbasin |
| 32. | Daffo | 2 | 9.2542 | 8.89125 | 1276 m | Seasonal endorheic drainage system on southern Jos Plateau |
| 33. | Daffo | 1 | 9.228717 | 8.85185 | 1285 m | Seasonal endorheic drainage system on southern Jos Plateau |
| 34. | Bokkos |  | 9.30965 | 8.989417 | 1113 m | Seasonal endorheic drainage system on southern Jos Plateau |
| 35. | Maijuju |  | 9.749722 | 9.151944 | 936 m | Trib. to eastern affluents Bagel and Gongola Rivers of Jos Plateau, Benue subbasin |
| 36. | Korot |  | 9.648367 | 8.945083 | 1223 m | Trib. to eastern affluents Maijuju and Gongola Rivers of Jos Plateau, Benue subbasin |
