# Supplementary material for: First DNA barcode library for the ichthyofauna of the Jos Plateau (Nigeria) with comments on potential undescribed fish species
Source: PeerJ. 2022 Apr 13;10:e13049. doi: 10.7717/peerj.13049 (PMC9013235; doi:10.7717/peerj.13049)
Supplement: Supplemental Information 10 — Known distribution ranges indicated by Locality Numbers in Table S1. [file peerj-10-13049-s010.docx]

| Order | Family | Genus | Species | Author/Comment | CO1 barcode available: | Distribution |
| --- | --- | --- | --- | --- | --- | --- |
| Cichliformes | Cichlidae | *Coptodon* | *zillii* (Morph I) | (Gervais, 1848) | Yes | 11, 13, 18, 28, 31. |
| Cichliformes | Cichlidae | *Coptodon* | *zillii* (Morph II) | (Gervais, 1848) | Yes | 1, 4, 10, 15, 17, 22, 27 28, 36. |
| Cichliformes | Cichlidae | *Oreochromis* | *niloticus* | Linnaeus, 1758 | Yes | 2, 13, 14, 23, 28, 36. |
| Cichliformes | Cichlidae | *Sarotherodon* | *galilaeus* | Linnaeus, 1758 | Yes | 14, 19, 24. |
| Cypriniformes | Cyprinidae | *Enteromius* | *perince* | (Rüppell, 1835) | Yes | 1, 5, 11, 12, 13, 15, 16, 18, 22, 29, 31, 33. |
| Cypriniformes | Cyprinidae | *Enteromius* | sp. Silver | **Unconfirmed candidate species** | Yes | 1, 2, 3, 4, 5, 6, 7, 8, 9, 10, 11, 12, 13, 14, 15, 16, 17, 18, 19, 22, 23, 24, 27, 29, 31, 32, 33. |
| Cypriniformes | Cyprinidae | *Enteromius* | sp. Gold | **Unconfirmed candidate species** | Yes | 1, 5, 14, 15, 18, 19, 22, 29, 36. |
| Cypriniformes | Cyprinidae | *Garra* | *trewavasae* | Monod, 1950 | Yes | 1, 2, 3, 4, 5, 6, 7, 8, 9, 10, 14, 15, 16, 22. |
| Cypriniformes | Cyprinidae | *Labeobarbus* | *bynni* | (Fabricius, 1775) | Yes | 2 |
| Cypriniformes | Cyprinidae | *Labeobarbus* | sp. Assop | **Unconfirmed candidate species** | Yes | 4 |
| Cypriniformes | Cyprinidae | *Labeo* | *parvus* | **Unconfirmed candidate species** | Yes | 1, 2, 15, 16, 19, 23, 29, 31. |
| Cypriniformes | Cyprinidae | *Labeo* | sp. Assop | **Unconfirmed candidate species** | Yes | 4 |
| Cypriniformes | Cyprinidae | *Raiamas* | *senegalensis* | Steindachner, 1870 | Yes | 2, 17, 23/2, 4, 14, 18, 24, 25. |
| Cypriniformes | Cyprinidae | *Raiamas* | *nigeriensis* | Daget, 1959 | Yes | 19, 24 and 2 |
| Cyprinodontiformes | Nothobranchidae | *Fundulopanchax* | *gardneri* | Boulenger, 1911 | Yes | 4, 5, 11. |
| Cyprinodontiformes | Poecilidae | *Poecilia* | *reticulata* | Peters, 1859 | Yes | 29 |
| Characiformes | Alestidae | *Brycinus* | *nurse* | Rüppell, 1832 | Yes | 2 |
| Osteoglossifromes | Mormyridae | *Mormyrus* | *hasselquistii* | Valenciennes, 1847 | Yes | 2 |
| Osteoglossifromes | Mormyridae | *Marcusenius* | *mento* | Boulenger, 1890 | No | 2 |
| Siluriformes | Mochokidae | *Chiloglanis* | *cf. benuensis* | Daget & Stauch, 1963 | No | 2 |
| Siluriformes | Mochokidae | *Synodontis* | *violaceus* | Pellegrin, 1919 | Yes | 2 |
| Siluriformes | Clariidae | *Clarias* | cf. *gariepinus* | Burchell, 1822 | No | 14 |
| Siluriformes | Clariidae | *Clarias* | sp. White dots | **Potential new species** | Yes | 13, 17, 20, 23. |
| Siluriformes | Clariidae | *Heterobranchus* | *longifilis* | Valenciennes, 1840 | Yes | 4 |
