# Supplementary material for: First DNA barcode library for the ichthyofauna of the Jos Plateau (Nigeria) with comments on potential undescribed fish species
Source: PeerJ. 2022 Apr 13;10:e13049. doi: 10.7717/peerj.13049 (PMC9013235; doi:10.7717/peerj.13049)
Supplement: Supplemental Information 11 — TDS, total dissolved solids; BOD, biochemical oxygen demand; TOC, total organic carbon. Water samples were independently taken from the ichthyological collection sites. [file peerj-10-13049-s011.docx]

|  |  |  |  |  |  |  |  |  |  |  |  |
| --- | --- | --- | --- | --- | --- | --- | --- | --- | --- | --- | --- |
| Sampling site | Water Temp (^0^C) | Turbidity (NTU) | Ap-Color (Pt-Co) | Tru Color (Pt-Co) | pH | Mg^2+^ (mg/L) | SO_4_ (mg/L) | NO_3_ (mg/L) | TDS (mg/l) | BOD_5_ (mg/L) | Conductivity (uS/cm) |
| St1. Luye (Tahoss) | 21.8 | 24.3 | 267.9 | 97.3 | 7.7 | 2.1 | 46.9 | 0.6 | 42.5 | 3.2 | 63.70 |
| St2. Ta'Aje (Tahoss) | 23.0 | 26.5 | 321.2 | 65.3 | 7.7 | 2.9 | 55.2 | 0.3 | 25.5 | 1.5 | 42.50 |
| St3. Voss (Tahoss) | 23.3 | 25.4 | 310.5 | 97.3 | 7.7 | 2.1 | 41.5 | 0.7 | 34.3 | 2.0 | 51.57 |
| St4. Wang (Tahoss) | 24.8 | 18.9 | 246.6 | 44.0 | 7.8 | 5.8 | 45.3 | 0.6 | 67.2 | 1.7 | 101.27 |
| St5. Assop | 24.2 | 46.1 | 545.1 | 150.6 | 7.9 | 2.3 | 40.3 | 0.8 | 46.6 | 1.5 | 69.97 |
| St6. Assop-Falls | 24.2 | 46.1 | 523.8 | 150.6 | 8.0 | 3.9 | 44.4 | 0.9 | 46.5 | 2.7 | 69.83 |
| St7. Rukuba | 23.3 | 47.2 | 651.7 | 129.3 | 7.4 | 1.7 | 39.1 | 1.0 | 30.2 | 10.7 | 45.43 |
| St8. Rukuba | 25.3 | 36.3 | 385.2 | 97.3 | 7.7 | 2.0 | 40.3 | 1.0 | 43.8 | 4.2 | 65.90 |
| St9. Rukuba | 26.3 | 70.1 | 715.7 | 86.6 | 7.6 | 2.1 | 39.5 | 1.0 | 43.4 | 6.5 | 65.23 |
| St10. Rukuba | 24.9 | 41.7 | 438.5 | 107.9 | 7.7 | 1.5 | 39.5 | 0.8 | 39.2 | 4.4 | 62.13 |
| St11. Rukuba | 24.7 | 59.2 | 619.7 | 150.6 | 7.5 | 1.2 | 44.0 | 1.0 | 34.1 | 3.7 | 51.20 |
| St12. N’gell | 22.2 | 169.2 | 1739.3 | 491.8 | 7.9 | 1.1 | 28.7 | 2.1 | 87.9 | 4.1 | 132.20 |
| St13. N’gell | 21.5 | 14.5 | 182.6 | 44.0 | 8.0 | 0.9 | 51.5 | 1.0 | 123.3 | 3.2 | 185.57 |
| St14. N’gell | 22.1 | 129.9 | 1430.1 | 353.2 | 8.0 | 1.2 | 41.5 | 1.2 | 102.9 | 9.0 | 154.83 |
