# Supplementary material for: First DNA barcode library for the ichthyofauna of the Jos Plateau (Nigeria) with comments on potential undescribed fish species
Source: PeerJ. 2022 Apr 13;10:e13049. doi: 10.7717/peerj.13049 (PMC9013235; doi:10.7717/peerj.13049)
Supplement: Supplemental Information 12 [file peerj-10-13049-s012.docx]

| **Order** | **Family** | **Species** | **Number of barcode sequences** | **Barcode Index Number (BIN)** | **Mean intraspecific distance** | **Maximum intraspecific distance** | **BIN member count** | **Max Divergence**  **in BIN:** | **Nearest BIN URI** | **Nearest Species**  **on BOLD** | **Average genetic distance to nearest neighbour species** |
| --- | --- | --- | --- | --- | --- | --- | --- | --- | --- | --- | --- |
| Characiformes | Alestidae | *Brycinus nurse* | 2 | BOLD:AAI8453 | 0 | 0 | 22 [22 Public] | 1.12% (p-dist) | BOLD:AEC5244 | *Alestes* sp. [Egypt] | 8.56% (p-dist) |
| Cichliformes | Cichlidae | *Coptodon zillii* | 14 | BOLD:AAB9042 | 0.21 | 0.46 | 139 [110 Public] | 5.33% (p-dist) | BOLD:AEH1296 | *Coptodon zillii* | 3.42% (p-dist) |
| Cichliformes | Cichlidae | *Oreochromis niloticus* | 8 | BOLD:AAA6537 | 0 | 0 | 462  [308 Public] | 3.63% (p-dist) | BOLD:AEH8512 | *Oreochromis niloticus* | 4.23% (p-dist) |
| Cichliformes | Cichlidae | *Sarotherodon galilaeus* | 5 | BOLD:AAA6537 | 0.15 | 0.31 | 462  [308 Public] | 3.63% (p-dist) | BOLD:AEH8512 | *Oreochromis niloticus* | 4.23% (p-dist) |
| Cypriniformes | Cyprinidae | *Enteromius perince* | 5 | BOLD:ACR9287 | 0 | 0 | 5 [5 Public] | 0% (p-dist) | BOLD:AAW3158 | *Enteromius perince* | 4.21% (p-dist) |
| Cypriniformes | Cyprinidae | *Enteromius* sp. Gold | 13 | BOLD:ACR9412 | 0 | 0 | 13 [13 Public] | 0% (p-dist) | BOLD:AAI4863 | "*Barbus* sp." [Angola] | 7.85% (p-dist) |
| Cypriniformes | Cyprinidae | *Enteromius* sp. Silver | 38 | BOLD:ACR9413 | 0.05 | 0.46 | 38 [38 Public] | 0.46% (p-dist) | BOLD:ACV4113 | *Enteromius brazzai* | 8.65% (p-dist) |
| Cypriniformes | Cyprinidae | *Garra trewavasae* | 31 | BOLD:ACR9351 | 0.04 | 0.46 | 31 [31 Public] | 0.46% (p-dist) | BOLD:ACH7729 | *Garra congoensis* | 3.37% (p-dist) |
| Cypriniformes | Cyprinidae | *Labeo parvus* | 21 | BOLD:AAA4266 | 0.12 | 0.31 | 167 [156 Public] | 1.66% (p-dist) | BOLD:AAL6602 | *Labeo parvus* | 3.85% (p-dist) |
| Cypriniformes | Cyprinidae | *Labeo* sp. Assop | 1 | BOLD:ACR9419 | N/A | 0 | 1 [1 Public] | N.A. | BOLD:ACC3876 | *Labeo parvus* | 2.88% (p-dist) |
| Cypriniformes | Cyprinidae | *Labeobarbus bynni occidentalis* | 1 | BOLD:ACR9402 | N/A | 0 | 1 [1 Public] | N.A. | BOLD:AAA5958 | Labeobarbus altianalis | 2.4% (p-dist) |
| Cypriniformes | Cyprinidae | *Labeobarbus* sp. Assop | 2 | BOLD:ACR9057 | 0 | 0 | 2 [2 Public] | 0% (p-dist) | BOLD:AAI7638 | *Enteromius cf. aboinensis* | 3.14% (p-dist) |
| Cypriniformes | Cyprinidae | *Raiamas nigeriensis* | 4 | BOLD:ACR9172 | 0.08 | 0.15 | 4 [4 Public] | 0.15% (p-dist) | BOLD:AAF5614 | Raiamas batesii | 12.02% (p-dist) |
| Cypriniformes | Cyprinidae | *Raiamas senegalensis* | 13 | BOLD:ACR9171 | 0.13 | 0.31 | 16 [17 Public] | 1.15% (p-dist) | BOLD:AAF8447 | Raiamas salmolucius | 12.66% (p-dist) |
| Cyprinodontiformes | Nothobranchiidae | *Fundulopanchax gardneri* | 3 | BOLD:ACR9440 | 0.1 | 0.15 | 3 [3 Public] | 0.15% (p-dist) | BOLD:ABA0207 | *Fundulopanchax gardneri* | 4.15% (p-dist) |
| Cyprinodontiformes | Poeciliidae | *Poecilia reticulata* | 1 | BOLD:ACE3484 | N/A | 0 | 104 [81 Public] | 8.19% (p-dist) | BOLD:AAD1850 | *Poecilia* sp. | 1.77% (p-dist) |
| Osteoglossiformes | Mormyridae | *Mormyrus hasselquistii* | 2 | BOLD:AAL5815 | 0 | 0 | 15 [15 Public] | 0% (p-dist) | BOLD:AAF4847 | *Mormyrus kannume* | 4.01% (p-dist) |
| Siluriformes | Clariidae | *Clarias sp. White* dots | 8 | BOLD:AAJ1105 | 0 | 0 | 36 [33 Public] | 1% (p-dist) | BOLD:AAJ1106 | *Clarias sp*. [DRC] | 2.99% (p-dist) |
| Siluriformes | Clariidae | *Heterobranchus longifilis* | 1 | BOLD:AAM0293 | N/A | 0 | 5 [5 Public] | 2.61% (p-dist) | BOLD:AAE2447 | *Clarias ngamensis* | 7.21% (p-dist) |
| Siluriformes | Mochokidae | *Synodontis violaceus* | 1 | BOLD:ACH7500 | N/A | 0 | 7 [7 Public] | 0.37% (p-dist) | BOLD:AAL5722 | Synodontis sp. Nigeria | 3.37% (p-dist) |
